# Biofilm formation on three different endotracheal tubes: a prospective clinical trial

**DOI:** 10.1186/s13054-020-03092-1

**Published:** 2020-06-29

**Authors:** Hulda R. Thorarinsdottir, Thomas Kander, Anna Holmberg, Sarunas Petronis, Bengt Klarin

**Affiliations:** 1 Department of Clinical Sciences, Lund University, Lund, Sweden; 2 Division of Intensive and Perioperative Care, Skåne University Hospital, Getingevägen 4, SE-22185 Lund, Sweden; 3 Division of Infection Medicine, Department of Clinical Sciences, Lund University, Lund, Sweden; 4 Chemistry, Biomaterials and Textiles, RISE Research Institutes of Sweden, Borås, Sweden

**Keywords:** Intubation, Intratracheal, Biofilm, Critical illness, Silicones, Polyvinyl chloride, Alloys, Pneumonia, Ventilator-associated

## Abstract

**Background:**

Biofilm formation on endotracheal tubes (ETTs) is an early and frequent event in mechanically ventilated patients. The biofilm is believed to act as a reservoir for infecting microorganisms and thereby contribute to development and relapses of ventilator-associated pneumonia (VAP). Once a biofilm has formed on an ETT surface, it is difficult to eradicate. This clinical study aimed to compare biofilm formation on three widely used ETTs with different surface properties and to explore factors potentially predictive of biofilm formation.

**Methods:**

We compared the grade of biofilm formation on ETTs made of uncoated polyvinyl chloride (PVC), silicone-coated PVC, and PVC coated with noble metals after > 24 h of mechanical ventilation in critically ill patients. The comparison was based on scanning electron microscopy of ETT surfaces, biofilm grading, surveillance and biofilm cultures, and occurrence of VAP.

**Results:**

High-grade (score ≥ 7) biofilm formation on the ETTs was associated with development of VAP (OR 4.17 [95% CI 1.14–15.3], *p* = 0.031). Compared to uncoated PVC ETTs, the silicone-coated and noble-metal-coated PVC ETTs were independently associated with reduced high-grade biofilm formation (OR 0.18 [95% CI 0.06–0.59], *p* = 0.005, and OR 0.34 [95% CI 0.13–0.93], *p* = 0.036, respectively). No significant difference was observed between silicon-coated ETTs and noble-metal-coated ETTs (OR 0.54 [95% CI 0.17–1.65], *p* = 0.278). In 60% of the oropharyngeal cultures and 58% of the endotracheal cultures collected at intubation, the same microorganism was found in the ETT biofilm at extubation. In patients who developed VAP, the causative microbe remained in the biofilm in 56% of cases, despite appropriate antibiotic therapy. High-grade biofilm formation on ETTs was not predicted by either colonization with common VAP pathogens in surveillance cultures or duration of invasive ventilation.

**Conclusion:**

High-grade biofilm formation on ETTs was associated with development of VAP. Compared to the uncoated PVC ETTs, the silicone-coated and noble-metal-coated PVC ETTs were independently associated with reduced high-grade biofilm formation. Further research on methods to prevent, monitor, and manage biofilm occurrence is needed.

**Trial registration:**

ClinicalTrials.gov NCT02284438. Retrospectively registered on 21 October 2014.

## Background

Ventilator-associated pneumonia (VAP) results in prolonged length of hospitalization, higher rates of morbidity and mortality, and significantly higher treatment costs [1–4]. Despite the introduction of preventive strategies and modifications of endotracheal tubes (ETTs), the rate of VAPs remains substantial [5–7]. In recent years, increasing evidence has emerged that biofilm formation on the surfaces of ETTs is an important link in VAP pathogenesis [8–14]. Furthermore, such biofilms act as reservoirs for pathogens that are believed to contribute to VAP relapses [12].

A number of different ETT surfaces or materials that are active against microbial adhesion or viability have been developed, but only silver-coated ETTs have been subjected to multiple clinical trials that have shown some beneficial effects [15]. However, there are still impediments to widespread use, including concerns over antibiotic resistance and the relatively high costs. Two ETT materials that are used extensively are polyvinyl chloride (PVC) and silicone-coated (SC) PVC. To the best of our knowledge, these two materials have not been compared regarding biofilm formation in a clinical setting. Another ETT coated with a thin layer of a noble metal alloy (NbMC) containing silver, gold, and palladium (Bactiguard® AB, Sweden) has been on the market since 2013, and the manufacturer claims that this coating does not release any silver ions into the environment. Urinary catheters with this coating have been successful in reducing urinary tract infections [16], but the effectiveness of the coating has not been evaluated in intensive care settings.

The primary aim of the present clinical prospective observational study was to compare the two most widely used ETT materials on the market (i.e., uncoated PVC and silicone-coated PVC) and an NbMC PVC ETT by evaluating the grade of biofilm formation on the three different ETT surfaces. The secondary objective was to explore possible associations between patient characteristics, the development of VAP, and increased biofilm formation on the ETT surfaces. We hypothesized that the three ETT materials would differ with regard to the grade of biofilm formation and also that the biofilm grade, along with colonization with common VAP pathogens in the oropharynx and lower airways, would be correlated with the development of VAP.

## Methods

This clinical observational study was carried out at Skåne University Hospital in Lund, Sweden. The study protocol was reviewed and approved by the Regional Ethical Review Board in Lund (protocol 2013/583). Informed consent, including permission to collect and publish anonymous data, was obtained from all patients or their relatives. The manuscript was prepared according to the STROBE guidelines for observational studies [17].

### Patients and materials

We included patients 18 years and older who were admitted to our intensive care unit (ICU) and were expected to require invasive mechanical ventilation for at least 24 h. Patients were allowed to participate only once and were included during six separate time periods from February 2014 to April 2017. Depending on the period, patients were intubated on clinical indications with one of the three different types of ETTs tested in our study. Each type of ETT was used in two of the six periods. The use of the different ETTs according to study period rather than by randomization was done for logistical reasons. Patients in study periods one and four received an uncoated PVC ETT (Oral/Nasal Endotracheal Tube, Mallinckrodt™, Medtronic, Dublin, Ireland), which is standard at our hospital; patients in periods two and six received an SC ETT (Siliconized PVC, Oral/Nasal Soft Seal® Cuffed Tracheal Tube, Portex™, Smith’s Medical, Kista, Sweden); and patients in periods three and five received an NbMC PVC ETT with a thin noble metal alloy coating consisting of gold, silver, and palladium (Bactiguard Infection Protection Endotracheal Tube, Bactiguard®, Tullinge, Sweden). Each of the six study periods was planned to last for about 3 months, and the goal was to include a minimum of 15 patients in each period. Bundles of maneuvers to prevent VAP (e.g., placing patients in a semi-recumbent position and controlling cuff pressure every 8 h to ensure a pressure of 20–30 mmHg) are standard at our facility. With the exception of use of different ETT materials during the study periods, all patients received standard intensive care according to their diagnosis and the clinical decisions of the responsible physicians. Furthermore, all patients were monitored daily for culture response, and a specialist in infection medicine who was blinded to the study evaluated antibiotic treatment daily based on culture response and proven experience.

### Microbiological procedures

Samples for surveillance cultures (i.e., oropharyngeal swabs and endotracheal aspirates) were collected from all included patients on days 1, 2, 3, 5, 7, 14, and 21 and thereafter once a week. This was also done on the day of extubation if not previously scheduled. All oropharyngeal swabs and endotracheal aspirates were processed in the same manner at the Department of Clinical Microbiology by standardized, extended microbiological procedures [18]. For details, please see the online supplementary material.

### Patient data

Age, sex, body mass index (BMI), days with invasive ventilation, antibiotic and/or proton pump inhibitor (PPI) use during mechanical ventilation, Simplified Acute Physiology Score 3 (SAPS 3), and main diagnosis on ICU admission were documented for all patients. Data on the occurrence of VAP and on microorganisms isolated in surveillance cultures and ETT biofilms were collected for all patients.

### Processing of the ETTs

Patients were extubated at the discretion of the treating physician or in the case of a patient’s death. After extubation, the ETTs were collected, avoiding contamination from other than oropharyngeal flora, and rinsed (inside and outside) with 1 L of sterile saline to eliminate excess mucus. Thereafter, the distal tip of the ETT was divided into four pieces for scanning electron microscopy (SEM; two pieces) and microbial cultures (two pieces). Finally, the ETT tip was cut in a cross-sectional manner 1.5 cm above the distal tip. Pieces of the ETT for microbial cultures were sonicated in phosphate-buffered saline (PBS) at 47 kHz for 90 s to dislodge biofilm microorganisms. The solution was then homogenized by vortex mixing and subsequently cultured using the same procedures as applied for the oropharyngeal swabs and endotracheal aspirates. An unpublished pilot study comparing different methods for processing of the ETTs had indicated that the method outlined above was optimal for removing the biofilm and for dislodging the microorganisms in the film before culturing. For SEM, pieces of an ETT were fixed in a solution of 4% formaldehyde diluted with PBS at room temperature for 30 min and then dehydrated with crescent ethanol concentrations, air-dried overnight, and sputter-coated with a thin (15-nm) Au/Pd film (Gatan PECS Mod 682, Gatan, Inc., Pleasanton, CA, USA) to prevent charging during SEM analysis.

### SEM and grading of the biofilm

The inner and outer surfaces of the ETTs were examined by SEM (Zeiss Supra 40VP, Carl Zeiss Microscopy GmbH, Jena, Germany). This analysis was performed using a secondary electron detector set at a working distance of 8–10 mm and electron acceleration of 3.68–4.05 kV. Low magnification (100× to 1000×) was used to rank biofilm coverage as follows: 0, no biofilm; 1, scarce coverage of < 10%; 2, clusters with 10–70% coverage; 3, confluent film with > 70% coverage. High magnification (10,000× to 50,000×) was used to evaluate biofilm density (0, no biofilm; 1, low/very porous; 2 medium; 3, high/compact) and level of thickness (0, no biofilm; 1, thin 0.1–1.0 μm; 2, medium 1.1–7 μm; 3, thick > 7 μm). Film thickness was estimated as the difference in focus distance between the outer surface of the biofilm and the ETT surface. When measuring the thickness, the mean was calculated based on multiple representative points in the sample. The final grade of the biofilm was then calculated by adding together the scores from coverage, density, and thickness to give a total score of 0 to 9. A high/advanced biofilm grade was defined as having a score of ≥ 7. The grading system is summarized in Table 1. Grading of the biofilm was performed by a researcher at RISE who was blinded to all patient information including type of ETT analyzed.

**Table 1:** System used to grade biofilm formation

Biofilm coverage	Biofilm density	Biofilm thickness scale	Biofilm grade
Determined at 100× to 1000× mag	Determined at 10,000× to 30,000× mag	Determined at 10,000× to 50,000× mag	**(coverage + density + thickness)**
0 no biofilm 1 scarce (< 10% coverage) 2 clusters (10–70% coverage) 3 confluent (> 70% coverage)	0 no biofilm 1 low/very porous 2 intermediate 3 high/compact	0 no biofilm 1 thin (0.1–1.0 mm) 2 medium (1.1–7 mm) 3 thick (> 7 mm)	0 no biofilm 1–3 low grade 4–6 medium grade 7–9 high grade

The biofilm grade was calculated by adding together the scores for biofilm coverage, density, and thickness. *Mag* magnification

### Definitions

Surveillance cultures and ETT tip cultures with growth of at least two species of bacteria commonly found in the oral cavity were classified as having normal flora (negative cultures). Species not normally found in the oral cavity (e.g., pathogens, gut flora, or overgrowth of normal oral flora) were classified as abnormal flora (positive cultures). All culture results were reviewed by a microbiologist to ensure correct classification. The diagnosis of VAP was made by two independent physicians evaluating the cases based on clinical and radiological examinations. VAP was defined by the following: (1) new or progressive lobar infiltrate > 48 h after intubation; (2) two or more of the minor criteria fever, leukocytosis/leukopenia, and purulent respiratory secretions; and (3) microbiological confirmation in endotracheal aspirate [19]. Colonization with common VAP pathogens included cultures with *Enterococcus faecium*, *E. faecalis*, *Staphylococcus aureus*, *Klebsiella pneumoniae*, *Acinetobacter baumannii*, *Pseudomonas aeruginosa*, *Streptococcus pneumoniae*, *Haemophilus influenzae*, and *other Enterobacteriaceae species.*

VAP relapse was defined as previously reported [12]. Microbial persistence was defined as persistence of the causative agent of VAP in at least two surveillance cultures despite 48 h of appropriate antibiotic therapy [20].

### Statistical analysis

Results were expressed as median (interquartile range [q1–q3]) for continuous variables and as numbers (percentages) for dichotomous variables. A *p* value of < 0.05 was considered significant, and all statistical tests were two-tailed. Fisher’s exact test for categorical variables was used for groupwise comparison of ETT cultures. Multivariable logistic regression analysis was applied to identify independent factors associated with the formation of high-grade biofilm on the ETT, that is, a score of ≥ 7 in the scoring system described above, and this cut-off was chosen based on data from an earlier study [11]. Univariable logistic regression analyses were conducted to evaluate patients’ characteristics that could be associated with the development of VAP. Multivariable regression analysis of the outcome VAP was not performed due to too few cases of the condition (*n* = 12). The Hosmer-Lemeshow test was used to determine goodness of fit for all logistic regression analyses. Clinical studies comparing the degree of biofilm formation on ETTs using the same scoring system applied in our investigation are lacking, and therefore, we based the present power calculations on previously published laboratory studies [21, 22]. Given a power of 0.8 and an alpha level of 0.05, a sample size of 27 was needed in each group. We carried out all analyses with SPSS 26 (SPSS Inc., Chicago, IL, USA).

## Results

**Fig. 1:**
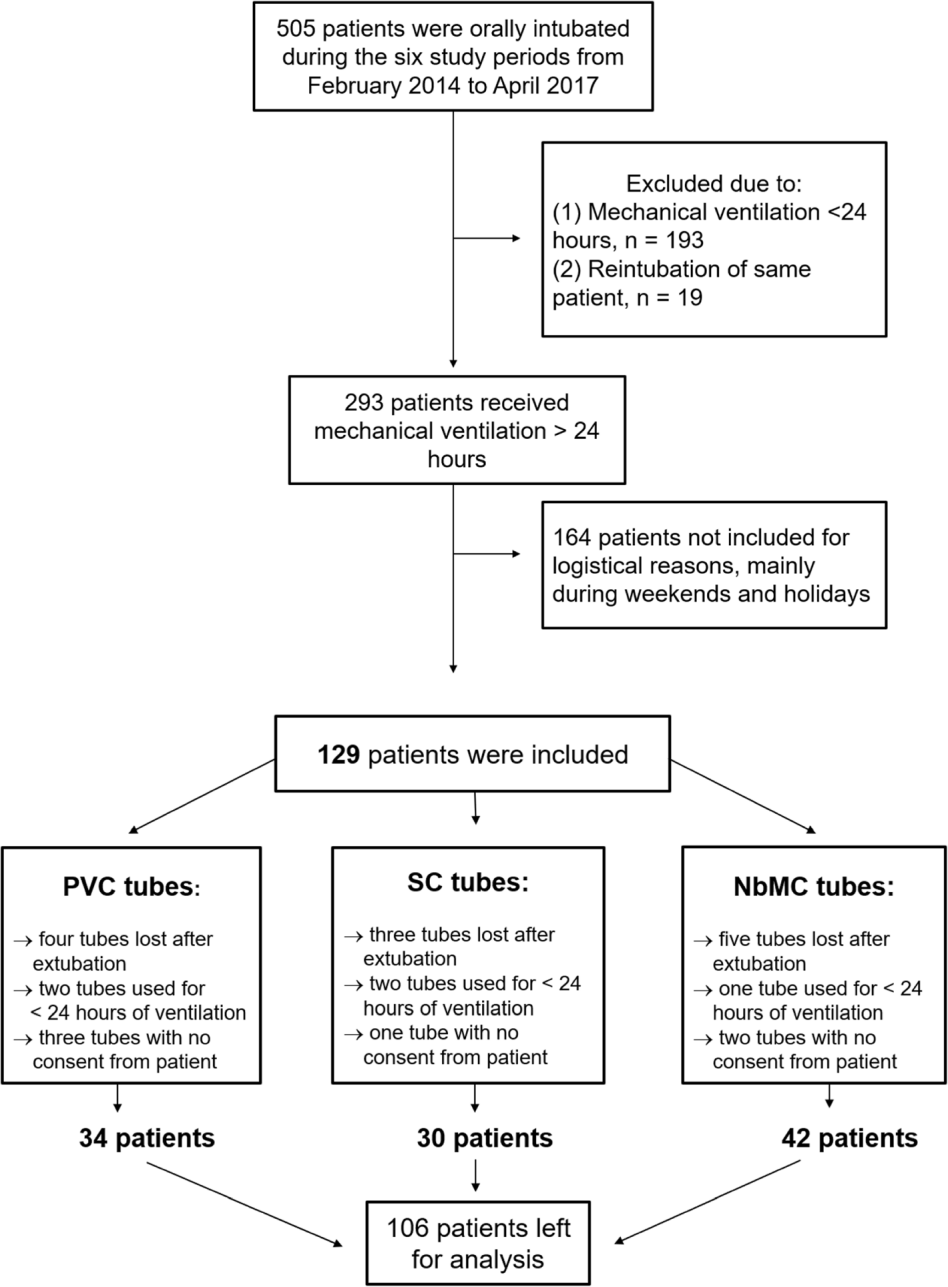
Flow chart showing inclusion of patients in the study. “No consent” refers to cases in which the patient did not give consent to participate after the ICU stay. Endotracheal tube materials: *PVC* uncoated polyvinyl chloride; *SC* silicon-coated PVC; *NbMC* noble-metal-coated PVC

Inclusion of patients is shown in Fig. 1. During the six study periods, 505 patients were orally intubated. One hundred twenty-nine of the 293 patients receiving mechanical ventilation for > 24 h were included in our study (Fig. 1). One hundred and sixty-four patients were not included for logistical reasons or during weekends and holidays. Twenty-three of the 129 patients that were originally included were subsequently excluded, because the ETT was lost after extubation, mechanical ventilation lasted < 24 h, and/or informed consent was not obtained from the patient or a relative. The remaining 106 patients were included in the final analysis as follows (Fig. 1): 34 were intubated with an uncoated PVC tube, 30 were intubated with an SC PVC tube, and 42 were intubated with the NbMC PVC tube. Patient characteristics and main diagnoses on ICU admission were similar in the three study groups (Table 2).

**Table 2:** Baseline characteristics of patients and diagnosis on admission to the ICU (*n* = 106)

Variable	PVC tube (***n*** = 34)	SC tube (***n*** = 30)	NbMC tube (***n*** = 42)
**Patient characteristics**			
Age, median (range)	67 (57–74)	69 (54–76)	69 (57–76)
Sex, male	16 (47)	17 (57)	27 (64)
BMI	26 (23–29)	28 (24–32)	26 (23–31)
SAPS III score	67 (53–75)	63 (57–79)	64 (52–74)
PPI use ≥ 24 h before intubation	19 (56)	13 (43)	24 (57)
PPI use while intubated	32 (94)	29 (97)	38 (91)
Antibiotic use ≥ 24 h before intubation	28 (82)	22 (73)	30 (71)
Antibiotic use while intubated	33 (97)	28 (93)	42 (100)
Days with invasive ventilation	3.3 (2.0–5.5)	3.0 (1.8–6.8)	3.6 (2.3–7.0)
**Main diagnosis on admission to the ICU**			
Sepsis	8 (23)	12 (40)	14 (33)
Cardiovascular	5 (15)	1 (3.3)	3 (7.1)
Respiratory insufficiency	11 (32)	13 (43)	19 (45)
Trauma	4 (12)	1 (3.3)	1 (2.4)
Coma GCS < 7	4 (12)	1 (3.3)	3 (7.1)
Other	2 (5.8)	2 (6.7)	2 (4.8)

Data are presented as median (q1–q3) or n (%). *Abbreviations: ICU* intensive care unit, *PVC* polyvinyl chloride, *SC* silicone-coated, *NbMC* noble-metal-coated, *BMI* body mass index, *SAPS III* Simplified Acute Physiology Score III, *PPI* proton pump inhibitor, *GCS* Glasgow Coma Scale

### Biofilm formation

**Fig. 2:**
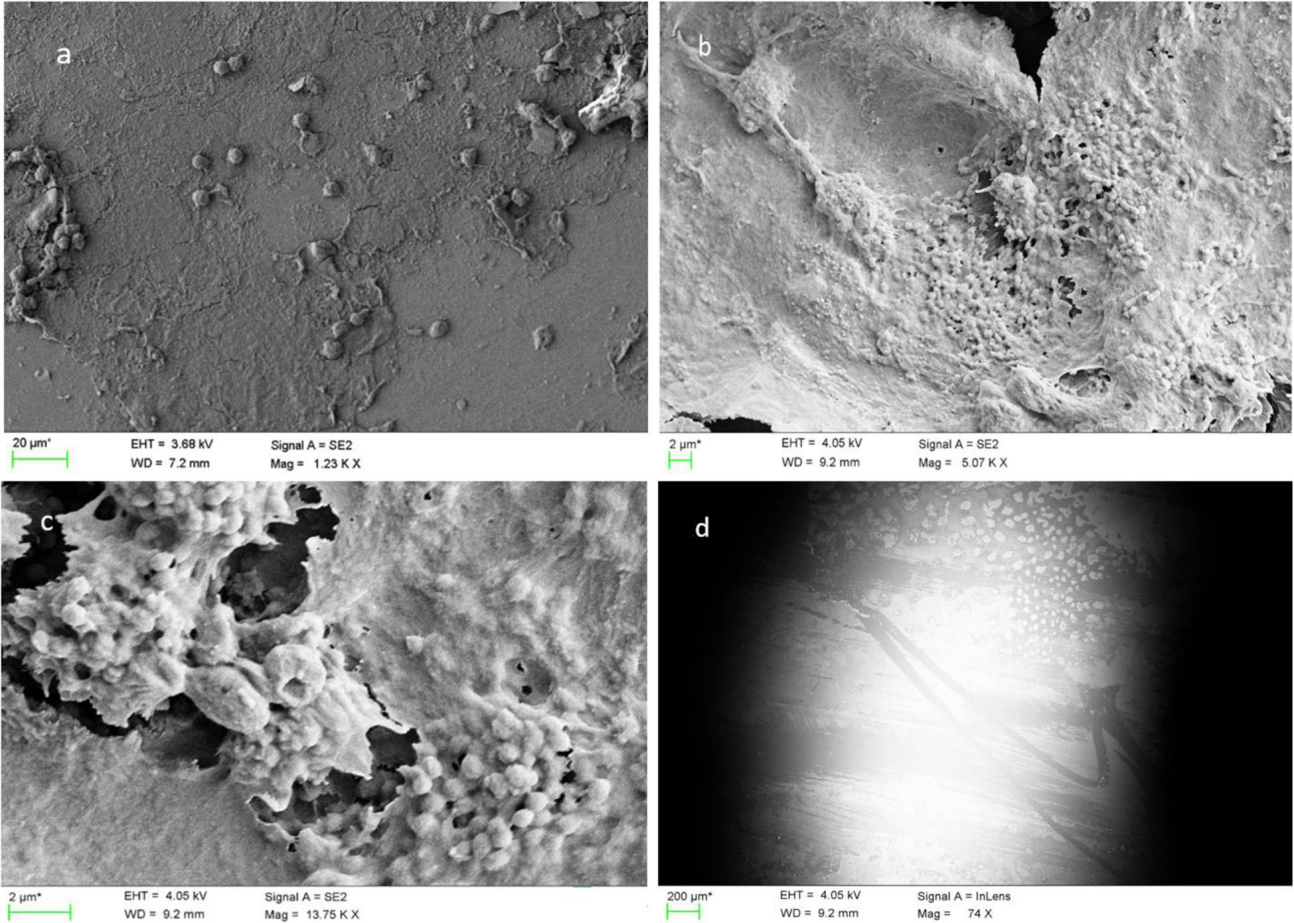
Scanning electron microscopy of biofilm formation on the surface of endotracheal tubes. **a** Typical low-grade (score < 4) biofilm formation. **b** Typical high-grade (score ≥ 7) biofilm formation (low magnification). **c** Colonies of microorganisms embedded in biofilm matrix (high magnification). **d** Scrape marks on the surface of an endotracheal tube probably caused by use of a suction catheter

Biofilm was present on the ETTs of 97% (*n* = 103) of the patients and could be seen on the surface of the tubes after 24 h of intubation. The biofilm formation varied from low/porous (Fig. 2a) to confluent/abundant biofilm matrices (Fig. 2b). Colonies of different microorganisms embedded in the biofilm matrix were often recognized by SEM (Fig. 2c).

When using multivariable logistic regression analysis to assess possible predictors of high-grade biofilm formation (score ≥ 7) on the ETTs, the SC ETTs and the NbMC ETTs were independently associated with reduced high-grade biofilm formation compared to the uncoated PVC tubes (odds ratio [OR] 0.18, 95% confidence interval [CI] 0.06–0.59, *p* = 0.005 for the former, and OR 0.34, 95% CI 0.13–0.93, *p* = 0.036 for the latter). There was no significant difference between SC and NbMC ETTs (OR 0.54; 95% CI 0.17–1.65, *p* = 0.278). Age, sex, days with invasive ventilation, and colonization with common VAP pathogens in surveillance cultures did not predict higher biofilm formation in univariable or multivariable analyses (Table 3).

**Table 3:** Possible predictors of high-grade (score ≥ 7) biofilm formation on the endotracheal tube

Factor	No ***n*** = 65	Yes ***n*** = 41	Univariable analysis			Multivariable analysis		
			OR	95% CI	***p*** value	OR	95% CI	***p*** value
ETT type								
PVC (reference)	15 (23)	19 (46)	NA	NA	NA	NA	NA	NA
SC	23 (35)	7 (17)	0.24	0.08–0.71	0.010	0.18	0.06–0.59	0.005
NbMC	27 (42)	15 (37)	0.44	0.17–1.11	0.081	0.34	0.13–0.93	0.036
Age	69 (58–76)	66 (53–75)	0.98	0.95–1.00	0.088	0.98	0.95–1.02	0.388
Sex, male	34 (52)	26 (63)	1.58	0.71–3.52	0.262	1.78	0.72–4.39	0.211
Days with invasive ventilation	3.1 (2.0–5.8)	3.8 (2.0–9.2)	1.06	0.98–1.14	0.157	1.06	0.97–1.15	0.219
Colonized	33 (51)	18 (44)	0.76	0.35–1.67	0.491	0.59	0.25–1.43	0.245

Data are presented as median (range) or number (percentage). “Colonized” refers to patients colonized with common VAP pathogens in surveillance (oropharyngeal or endotracheal) cultures. *Abbreviations: OR* odds ratio, *CI* confidence interval, *ETT* endotracheal tube, *PVC* polyvinyl chloride, *SC* silicone-coated, *NbMC* noble-metal-coated, *VAP* ventilator-associated pneumonia, *NA* not applicable

### Surveillance cultures

Surveillance cultures were obtained for all patients, but cultures that were missing according to the predefined culture scheme represented 5% (*n* = 41) of all planned cultures. For all oropharyngeal (*n* = 376) and endotracheal (*n* = 381) cultures, 66% (*n* = 248) and 64% (*n* = 242), respectively, turned positive and about 22% of those were noted as polymicrobial. A majority of the patients developed abnormal oropharyngeal flora (82%) and became colonized in the lower airways (79%) during invasive ventilation. Colonization with common VAP pathogens was found in oropharyngeal cultures from 33% (*n* = 35) of the patients and in endotracheal cultures from 27% (*n* = 29). In colonized patients, the median time from intubation to culture positivity was 1 day for both oropharyngeal and endotracheal cultures. In patients colonized in oropharyngeal or in endotracheal cultures (all positive cultures), the same microorganism was found on the ETT in 48% and 49% of the cases, respectively. In patients colonized with common VAP pathogens, the same microorganism was found on the ETT in 29% (10 out of 35) of the oropharyngeal cultures and 38% (11 out of 29) of the endotracheal cultures.

Microorganisms isolated from surveillance cultures in the three study groups are listed in Table 4. The surveillance cultures turned positive for several different microorganisms, most often *Candida albicans* and other candida species. Among bacteria, *Enterococcus faecalis*, *E. faecium*, *Staphylococcus aureus*, *Klebsiella* species, *Stenotrophomonas maltophilia*, *Pseudomonas aeruginosa*, and *Haemophilus influenzae* occurred most frequently (Table 4).

**Table 4:** Microbial isolation from surveillance cultures in the three study groups

	OPC	OPC-ETT match	ETA	ETA-ETT match
**Total number of different microorganisms isolated**	**150 (100)**	**51 (100)**	**133 (100)**	**51 (100)**
**Microorganism**				
**Gram-positive bacteria**				
*Enterococcus faecalis*	15 (10)	4 (7.8)	10 (7.5)	4 (7.8)
*Enterococcus faecium*	13 (8.6)	2 (3.9)	5 (3.9)	1 (2.0)
*Staphylococcus aureus*	9 (6.0)	3 (5.9)	15 (11)	4 (7.8)
*Staphylococcus epidermitis*	2 (1.3)	1 (2.0)	2 (1.5)	
Beta hemolytic streptococcus group A	1 (0.7)		1 (0.7)	
*Streptococcus pneumoniae*			1 (0.7)	
**Gram-negative bacteria**				
*Klebsiella* species	9 (6.0)	1 (2.0)	4 (3.1)	2 (3.9)
*Stenotrophomonas maltophilia*	8 (5.2)	4 (7.8)	7 (5.3)	4 (7.8)
*Pseudomonas aeruginosa*	6 (4.0)	4 (7.8)	5 (3.9)	4 (7.8)
*Haemophilus influenzae*	4 (2.7)		5 (3.9)	
*Enterobacter cloacae*	2 (1.3)		2 (1.5)	
*Serratia marcescens*	3 (2.0)	1 (2.0)	1 (0.7)	1 (2.0)
Escherichia coli*	4 (2.7)	1 (2.0)	2 (1.5)	
*Citrobacter freundii*	2 (1.3)	1 (2.0)	1 (0.7)	1 (2.0)
*Chryseobacterium indologenes*	2 (1.3)	2 (3.9)	3 (2.3)	2 (3.9)
*Acinetobacter* sp.	1 (0.7)		1 (0.7)	
**Other bacterial species**	5 (3.4)		8 (6.2)	
**Yeast**				
*Candida* species	60 (40)	27 (53)	52 (39)	27 (53)
*Candida albicans*	41 (27)	21 (41)	37 (28)	21 (41)
*Candida dubliniensis*	8 (5.3)	1 (2.0)	6 (4.5)	1 (2.0)
*Candida parapsilosis*	2 (1.3)	1 (2.0)	2 (1.5)	1 (2.0)
*Candida tropicalis*	2 (1.3)	1 (2.0)	2 (1.5)	1 (2.0)
Other candida species	7 (4.7)	3 (5.9)	5 (3.9)	3 (5.9)
Other yeasts, not candida	4 (2.7)		8 (6.0)	1 (2.0)

*Note:* Data are presented as numbers (percentages). The percentage of positive cultures is related to how many patients were colonized with the specific microorganism. *Includes *Escherichia coli* producing extended-spectrum beta-lactamase (ESBL)*.* Approximately 25% of oropharyngeal cultures and 18% of endotracheal cultures were polymicrobial. *Abbreviations: OPC* oropharyngeal culture, *ETA* endotracheal culture, *ETT* endotracheal tube

### Microbial isolation from endotracheal tubes

At extubation, 99% (*n* = 105) of the ETTs were cultured and 47% (*n* = 49) turned positive. No significant difference in positive ETT cultures was observed between the groups (uncoated PVC 41% [*n* = 14], SC PVC 45% [*n* = 13], and NbMC PVC 52% [*n* = 22]; *p* = 0.61). An ETT culture positive for common VAP pathogens at extubation was predicted by colonization with common VAP pathogens in oropharyngeal cultures (OR 17.6 [95% CI 3.8–81.6], *p* < 0.001) and in endotracheal cultures (OR 10.3 [95% CI 3.1–34.2], *p* < 0.001). If the ETT tip turned positive, the microorganism was found in oropharyngeal cultures in 86% (*n* = 42) of the patients and in endotracheal cultures in 88% (*n* = 43). The microorganisms found on the ETTs at points of extubation could be detected in the first oropharyngeal culture in 60% (*n* = 29) of the patients and in the first endotracheal cultures in 58% (*n* = 28). *Candida albicans* (39%) and other candida species (11.5%) were the microorganisms that occurred most frequently on the ETTs, followed by *Staphylococcus aureus* (8.2%), *Enterococcus faecalis* (8.2%), *Pseudomonas aeruginosa* (6.6%), and *Stenotrophomonas maltophilia* (6.6%). Microbial isolations from the different ETTs are presented in Table 5.

**Table 5:** Microbial isolation from positive endotracheal tube tip cultures

Organism	PVC tube (*n* = 14)	SC tube (*n* = 13)	NbMC tube (*n* = 22)	All tubes (*n* = 49)
**Gram-positive bacteria**				
*Staphylococcus aureus*	2 (11)	2 (13)	1 (3.7)	5 (8.2)
*Enterococcus faecalis*	3 (16)		2 (7.4)	5 (8.2)
*Enterococcus faecium*	1 (5.6)	1 (6.2)		2 (3.3)
*Staphylococcus epidermidis*		1 (6.3)		1 (1.6)
**Gram-negative bacteria**				
*Pseudomonas aeruginosa*	1 (5.6)		3 (11)	4 (6.6)
*Stenotrophomonas maltophilia*			4 (15)	4 (6.6)
*Klebsiella* species		1 (6.2)	1 (3.7)	2 (3.3)
*Chryseobacterium indologenes*			2 (7.4)	2 (3.3)
*Escherichia coli*		1 (6.2)		1 (1.6)
*Serratia marcescens*	1 (5.6)			1 (1.6)
*Acinetobacter* species		1 (6.2)		1 (1.6)
*Citrobacter freundii*			1 (3.7)	1 (1.6)
**Yeast**				
*Candida* spp.	9 (50)	9 (56)	13 (48)	31 (51)
*Candida albicans*	8 (44)	7 (44)	9 (33)	24 (39)
Other yeasts	1 (5.6)			1 (1.6)
**Total number of microorganisms cultured from ETTs**	**18 (100)**	**16 (100)**	**27 (100)**	**61 (100)**

Data are presented as number (percentage). Approximately 20% of all endotracheal tube tips were polymicrobial. *Abbreviations: PVC* polyvinyl-chloride, *SC* silicone-coated, *NbMC* noble-metal-coated

### VAP

Twelve patients developed 15 episodes of VAP during their stay in the ICU, and three of those episodes were VAP relapses. The most common pathogens involved in VAP were *Enterococcus faecium*, *E. faecalis*, *Staphylococcus aureus*, *Klebsiella* spp., and *Acinetobacter* spp. High-grade biofilm formation (score ≥ 7) on the ETTs, days of invasive ventilation, and age were significantly associated with the development of VAP (OR 4.17, 95% CI 1.14–15.3, *p* = 0.031; and OR 1.11, 95% CI 1.01–1.22, *p* = 0.026; and OR 0.96, 95% CI 0.92–0.99, *p* = 0.046, respectively). ETT material, sex, and colonization with common VAP pathogens in surveillance cultures were not associated with development of VAP (Table 6).

**Table 6:** Analysis of possible predictors of ventilator-associated pneumonia (*n* = 83)

	Univariable analysis		
Factor	OR	95% CI	***p*** value
ETT type			
PVC (reference)	NA	NA	NA
SC	0.66	0.14–3.16	0.606
NbMC	0.54	0.13–2.26	0.400
Age	0.96	0.92–0.99	0.046
Sex, male	0.68	0.19–2.48	0.563
Days with invasive ventilation	1.11	1.01–1.22	0.026
High-grade biofilm formation on ETT	4.17	1.14–15.3	0.031
Colonized	1.52	0.44–5.26	0.505

“Colonized” refers to patients colonized with common VAP pathogens in surveillance (oropharyngeal or endotracheal) cultures. *Abbreviations: OR* odds ratio, *CI* confidence interval, *ETT* endotracheal tube, *PVC* polyvinyl chloride, *SC* silicone-coated, *NbMC* noble-metal-coated, *NA* not applicable

Microbial persistence in surveillance cultures could be evaluated in seven of 12 patients who developed VAP, and it occurred in five patients (71%) after appropriate antibiotic treatment for VAP. At extubation, the microorganisms previously causing VAP could be found in the ETT biofilm in 56% of the cases (5 out of 9 patients) despite appropriate antibiotic therapy. The microorganisms most often involved in microbial persistence were *Klebsiella* spp., *Candida parapilosis*, *Enterococcus faecium*, and *E. faecalis*. In about half of the VAP cases, an evaluation of microbial persistence in surveillance cultures (*n* = 5) or in the ETT biofilm (*n* = 3) could not be done, because the course of antimicrobial treatment (primarily antifungal treatment < 7 days) was too short, the pathogens were resistant to initial treatment (inappropriate antibiotic therapy), or the patient received a tracheostomy or died.

## Discussion

The results of this prospective clinical observational study suggest that high-grade biofilm formation on ETTs is associated with the development of VAP. Of the three ETT materials, we investigated, both the SC ETT and the NbMC ETT were associated with reduced high-grade biofilm formation compared to the standard uncoated PVC tube. The microorganisms detected in the ETT biofilm at extubation were frequently found in surveillance cultures at intubation. In patients who developed VAP, the causative microbe often remained in the biofilm despite appropriate antibiotic therapy. High-grade biofilm formation on ETTs was not predicted by either colonization with common VAP pathogens in surveillance cultures or duration of invasive ventilation.

The determinants of high-grade biofilm formation on ETTs in critically ill patients are not clear and are probably multifactorial. In this study, we found that high-grade biofilm formation on ETTs was associated with the development of VAP, and this has also been observed in a previous assessment including 32 patients [11]. Our investigation confirms this finding but in a much larger population (*n* = 106). Unfortunately, there is no gold standard for biofilm grading when using SEM, which makes it difficult to compare results from different studies. The majority of assessments that have performed SEM to analyze biofilms have graded biofilm formation solely by considering the coverage or the existence of a biofilm [12, 23–25]. The disadvantage of that approach is that two biofilms exhibiting the same coverage can differ substantially in thickness and density, and most likely also with regard to their degree of maturity and how prone they are to dispersal [26]. This may explain why the authors of some earlier studies have not found an association between biofilm coverage/existence and VAP development [12, 27].

Although biofilm formation is a rapid process, we were unable to predict biofilm grade solely by determining the duration of invasive ventilation. This has also been seen in previous studies, which indicates that the grade of biofilm formation is dependent on additional factors [8, 11, 12]. It is not clear what causes high-grade biofilm to rapidly develop on the ETT surface in one patient, but to appear much more slowly or not at all in another. Similarly, not all intubated patients ultimately develop VAP [11]. Being able to monitor or predict the grade of biofilm formation is of clinical importance, because our findings indicate that high-grade biofilm formation is associated with the development of VAP. Methods for continuous monitoring of biofilm formation on ETTs have been described in laboratory models in which optical fiber sensors are incorporated in the lumen of the ETT [28]. This approach represents an interesting tool for clinical use, although it must first be evaluated in such a setting. Another promising strategy that might be further investigated is biofilm removal without ETT removal by use of tools such as the mucus shaver or photodynamic inactivation [29, 30].

Despite the knowledge that VAP is the most common hospital-acquired infection in the critically ill, and that ETT biofilms act as a significant and persistent source of infection in intubated patients, routines for biofilm removal including ETT exchange are not well studied and in many cases are not considered safe. Inasmuch as biofilms are difficult to eradicate once they have formed, it would be interesting to evaluate whether changing the ETT in selected VAP patients could reduce microbial persistence and the risk of VAP relapse. The risk-to-benefit ratio must be evaluated for each individual patient, but the benefits of an ETT change may be greater than the risks in some cases. It has been pointed out that reintubation is associated with VAP, although some of the data underlying that conclusion have been obtained in extubation trials rather than in evaluations of ETT exchange [31]. Nevertheless, well-controlled studies in this field are warranted before any recommendations can be made.

Considering the life cycle of a biofilm, it is likely that high-grade biofilm formation (grade ≥ 7 in our study) reflects a more mature biofilm containing several pillar- and mushroom-shaped masses that are susceptible to breakage caused by turbulent airflow or manipulation such as suction with a catheter (as shown in Fig. 2d). Detachment and dispersal are natural developments in a mature biofilm that lead to the spread of highly contagious fragments of the biofilm into the lower airways [32]. On the other hand, a low-grade biofilm is thinner and more firmly anchored to an ETT surface when host- and tissue-specific adhesins on pili and fimbria are attached [33].

Colonization with common VAP pathogens in surveillance cultures was not associated with high-grade biofilm formation or VAP in our study. This finding was somewhat unexpected, considering that colonization with pathogenic bacteria is assumed to precede the development of VAP [34, 35]. Previous research has shown that there is a microbial link between oropharyngeal, tracheal, and biofilm cultures [12], but, to the best of our knowledge, the correlation between positive cultures and biofilm grade has not yet been elucidated. Vandecandelaere et al. used both culture and culture-independent methods and found no significant difference in biofilm flora between patients who developed VAP and those who did not [36]. Furthermore, those authors observed no difference in biofilm flora between patients with longer (> 5 days) and shorter (< 5 days) intubation periods. It has been suggested that many microorganisms in the oral flora initiate biofilm formation that may facilitate colonization of more pathogenic bacteria, although those floral microorganisms may not be responsible for development of VAP per se [37]. The impact of each microorganism or combination of microorganisms is not clear and must be elucidated in larger trials.

This is the first study of critically ill patients to compare biofilm formation on widely used ETT materials. We found that, compared to the standard uncoated PVC ETT, the SC ETT and the NbMC ETT were associated with reduced high-grade biofilm formation. Although silicone is applied extensively in health care, there are no previous evaluations of the use of SC ETTs in the critical care setting. It should be mentioned that there are a number of subtypes of silicone elastomers, and the exact composition of the particular subtype used in the present SC ETTs is the manufacturer’s trade secret and hence was not available to us.

Different catheter materials have been investigated in vitro, and although results are conflicting, some of the authors have reported significantly reduced bacterial adhesion and biofilm formation on silicone surfaces compared to PVC surfaces [21, 38]. Also, studies have demonstrated that the properties of PVC and silicone catheters from different manufacturers vary, indicating differences in the composition of the materials [22]. Hence, our findings may not apply to all silicone or PVC ETTs. It has also been observed that biofilm formation is facilitated on surfaces with greater roughness [39]. Even though evaluation of surface topography was not one of the objectives of our study, we did examine the surfaces of unused ETTs by SEM and found no subjective signs of differences between the tube materials.

The NbMC ETTs have not been evaluated in the intensive care setting, with the exception of one short-term intubation trial performed during elective surgery [40]. In our study, no adverse reactions were registered that could be related to the NbMC ETT coating. Urinary catheters containing this coating have shown reduced catheter-related urinary tract infections with long-term use [41–43]. However, it should be noted that NbMC urinary catheters are made of silicone or latex, not PVC like the NbMC ETT in our study. PVC ETTs have been reported to be prone to bacterial adhesion in vitro [44], which indicates that silicone ETTs coated with noble metals may be more efficient in preventing microbial adhesion and biofilm formation. This possibility must be further assessed before any conclusions can be drawn.

We recognize the limitations of the present single-center study. First, our ambition was to include all patients that were expected to receive mechanical ventilation for > 24 h. For logistical reasons and during weekends and holidays, a number of patients were not included (*n* = 164). Nonetheless, patient characteristics in the three assessment groups are similar, and the same method of inclusion was applied to all six inclusion periods. The six study periods were also equally spread throughout the year, including some holiday periods and summer months. Another limitation is that one would expect to observe the same significant association between ETT material and VAP as is noted between ETT material and high-grade biofilm formation. However, not all patients with high-grade film formation on the ETT surface can be expected to develop VAP; according to our analyses, the odds are only increased. One probable explanation for this is lack of power, because a much larger sample size is needed to detect a difference in VAP than to identify a change in grade of biofilm formation. Although bundles of maneuvers to prevent VAP are standard at our facility, information on cuff pressure and head of bed elevation could not be retrieved for individual patients. Also, the duration of antibiotic treatment could not be evaluated in this investigation.

## Conclusions

In this study, formation of biofilm on ETTs was an early and frequent event in critically ill patients. Moreover, high-grade biofilm formation on an ETT was associated with the development of VAP. Among the three tube materials we investigated, both the SC ETT and the NbMC ETT were associated with reduced high-grade biofilm formation as compared to the standard uncoated PVC ETT. The microorganisms detected in the ETT biofilm at extubation were frequently found in surveillance cultures at intubation. In patients who developed VAP, the causative microbe often remained in the biofilm despite appropriate antibiotic therapy. High-grade biofilm formation on ETTs was not predicted by either colonization with common VAP pathogens in surveillance cultures or duration of invasive ventilation. Further research on methods to prevent, monitor, and manage the occurrence of biofilms on ETTs is required.

## Supplementary information

**Additional file 1.** Processing of oropharyngeal swabs and endotracheal aspirates

## Data Availability

The datasets used and/or analyzed in the current study are available from the corresponding author upon reasonable request.

## Abbreviations

ETT: Endotracheal tube
SEM: Scanning electron microscopy
HAP: Hospital-acquired pneumonia
ICU: Intensive care unit
MALDI-TOF: Matrix-assisted laser desorption/ionization time-of-flight
NbMC: Noble-metal-coated
PBS: Phosphate-buffered saline
PPI: Proton pump inhibitor
PVC: Polyvinyl chloride
SAPS 3: Simplified Acute Physiology Score 3
SC: Silicone-coated
VAP: Ventilator-associated pneumonia
