# Supplementary material for: Biofilm formation on three different endotracheal tubes: a prospective clinical trial
Source: Crit Care. 2020 Jun 29;24:382. doi: 10.1186/s13054-020-03092-1 (PMC7322705; doi:10.1186/s13054-020-03092-1)
Supplement: Supplementary file 1 — Additional file 1. Processing of oropharyngeal swabs and endotracheal aspirates [file 13054_2020_3092_MOESM1_ESM.docx]

**Processing of oropharyngeal swabs and endotracheal aspirates:**

Samples were inoculated on three different selective plates, one differentiating and one non-selective agar plate as follows: (1) agar plate with 5% horse blood (LabM, Heywood, Lancashire, UK) supplemented with 10 mg/L colistin and 15 mg/L nalidixic acid; (2) agar plate with 5% horse blood supplemented with 2 mg/L gentamicin and 25 mg/L nalidixic acid for Gram-positive cocci including *S. pneumoniae*; (3) Hematin agar plate (OxoidTM, Thermo Science, Basingstoke, UK) supplemented with 300 mg/L bacitracin for fastidious Gram-negative rods including *Haemophilus influenzae* (selective); (4) Uriselect 4 agar (Bio-Rad Laboratories, Copenhagen, Denmark) supplemented with 10 mg/L vancomycin for non-fastidious Gram-negative rods (differentiating); and (5) Hematin agar with a colistin disk (non-selective). All plates were manufactured in-house, and they were inspected for growth after 16 and 40 hours of aerobic, anaerobic, or CO2 incubation at 35 - 37 °C. Identification of bacterial species was performed using matrix-assisted laser desorption/ionization time-of-flight (MALDI-TOF) mass spectrometry (MALDI Biotyper Microbial Identification system, Bruker, Boston, MA, USA). Differentiation of *Candida* spp was based on colony appearance on CHROM Candida agar (CHROMagar, Hägersten, Sweden) after 48 hours of incubation at 35 °C.
